# Ventricular tachycardia of right ventricular outflow tract origin during the perioperative period: A case report

**DOI:** 10.1097/MD.0000000000026372

**Published:** 2021-06-25

**Authors:** Ki Hyun Park, Hyun Kyoung Lim, Na Eun Kim, Helen Ki Shinn, Yong Soo Baek

**Affiliations:** a Department of Anesthesiology and Pain Medicine, Inha University College of Medicine, 27, Inhang-ro, Jung-gu; b Division of Cardiology, Department of Internal Medicine, Inha University Hospital, Incheon, Republic of Korea.

**Keywords:** abdominal surgery, dopamine, ephedrine, monomorphic ventricular tachycardia, pacemaker, right ventricular outflow tract

## Abstract

**Rationale:**

Idiopathic ventricular tachycardia (VT) occurs in individuals without structural abnormalities in the heart, accounts for approximately 10% of total VTs. Furthermore, approximately 70% of idiopathic VTs originate from Right ventricular outflow tract (RVOT). **However, among perioperative arrhythmias, incidence of VT after surgery is extremely rare and most arrhythmias are atrial origin**.

**Patient concerns:**

A 69-year-old man with permanent pacemaker underwent colon surgery.

**Diagnoses:**

Patient suffered from low blood pressure and dizziness, sweating at post anesthetic care unit (PACU) and heart rate (HR) increased suddenly to 200 beats/min with monomorphic VT after bolus ephedrine administration and continuous dopamine infusion.

**Interventions:**

Pacemaker interrogation followed by DC cardioversion was done.

**Outcomes:**

Patient's vital signs became normal and symptoms are subsided.

**Lessons:**

RVOT VT can be caused by triggering activities, such as ephedrine, dopamine, and inadequate fluid management. These triggering activities are initiated by acceleration of HR from ventricles with infusion of catecholamine which lead monomorphic VT originating from RVOT.

RVOT origin PVCs can be precipitated into monomorphic VT by administrating catecholamines such as ephedrine and dopamine even in patient with pacemaker. The mechanism of these VTs includes catecholamine induced acceleration of HR. Since RVOT PVCs can be recognize by 12 EKGs, we should be pay more attentions to the pre-operation EKG and be cautious using catecholamines.

## 1 Introduction

As the number of patients with cardiac implantable electronic devices has recently increased, there are a number of cases where anesthesiologists and surgical teams need to make preparations related to this intervention before surgery. The preoperative management is performed according to the guidelines issued by the heart rhythm society in 2011.^[1]^

Newly developed postoperative arrhythmias in patients who underwent non-cardiac surgery occurs about 4% to 20%.^[2]^ However, among these arrhythmias, incidence of ventricular tachycardia (VT) from right ventricular outflow tract (RVOT) is extremely rare.

This report aimed to outline the management of a patient with a pacemaker implanted because of sick sinus syndrome (SSS) where RVOT - VT occurred in post anesthesia care unit (PACU).

## 2 Case description

**Figure 1:**
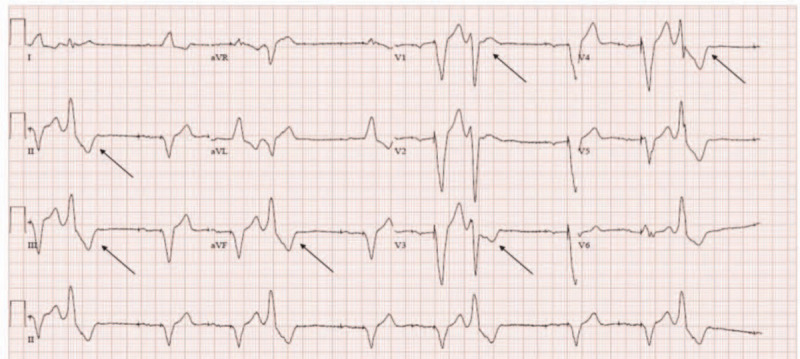
Preoperative 12-lead EKG; LBBB in V1, R/S transition in V3–4, and a tall R wave in leads II, III, and aVF which indicating PVCs from RVOT.

A 69-year-old man (body weight, 64. 4 kg; height, 156. 6 cm) was diagnosed with carcinoma of the ascending colon and scheduled to undergo laparoscopic right hemicolectomy and laparoscopic cholecystectomy. He had been diagnosed with hypertension a year ago and underwent pacemaker implantation due to SSS, complete AV block, and left bundle branch block (LBBB) in the same year. He had a dual-chamber pacemaker with atrial ventricular sensing, dual response, and rate-adaptive pacemaker (DDDR). In the preoperative evaluation, the electrocardiogram (EKG) revealed AV dual-paced rhythm with frequent premature ventricular contractions (PVCs). His heart rate (HR) was 68 beats/min, and QTc was 499 ms. PVC morphology has special characteristics of LBBB at V1, R/S transition in leads V3–4, and a tall R wave in leads II, III, and aVF, indicating PVC originating in the RVOT (Fig. 1).^[2]^ Transthoracic echocardiography showed normal-sized cardiac chambers with preserved LV global systolic function (LVEF 64%) and normal valvular structure and function. The patient's pulmonary artery pressure was 37 mmHg, with no regional wall motion abnormalities. Moreover, there was no specific finding on performing coronary angiography. An adrenal mass was noted on performing abdominal CT and pheochromocytoma was ruled out.

Preoperative laboratory investigation showed no specific findings, with a hemoglobin (Hgb) level of 9.8 g/dL, sodium level of 142 mEq/L, potassium level of 3.54 mEq/L, chloride level of 103 mEq/L, and calcium level of 9.6 mg/dL.

Elective surgery was performed, which lasted for 4 hours and 35 minutes. Total time of anesthesia was 5 hours and 10 minutes. Preoperatively, the pacemaker mode was changed from DDDR to DOO (asynchronous mode). General anesthesia was induced totally with intravenous propofol which has anti-arrhythmic effects and remifentanil. Before intubation, 40 mg of lidocaine was injected intravenously, and 40 mg of rocuronium was used as a muscle relaxant. During the operation, the target concentration of anesthetics was maintained with propofol 2.5 to 3.0 mcg/mL, remifentanil 1 to 3 ng/mL, and rocuronium 5 mcg/kg/min. During the emergence, glycopyrrolate 0.2 mg, pyridostigmine 10 mg, and sugammadex 200 mg were administered. The vital signs of the patient were stable during surgery; frequent PVC was not observed, and his blood pressure was between 100 to 130 and 60 to 90 mmHg. His HR was maintained at 60 beats/min. The total estimated blood loss was approximately 400 mL, total urine output was 630 mL, and total administered fluid volume was 2100 mL of balanced crystalloids.

**Figure 2:**
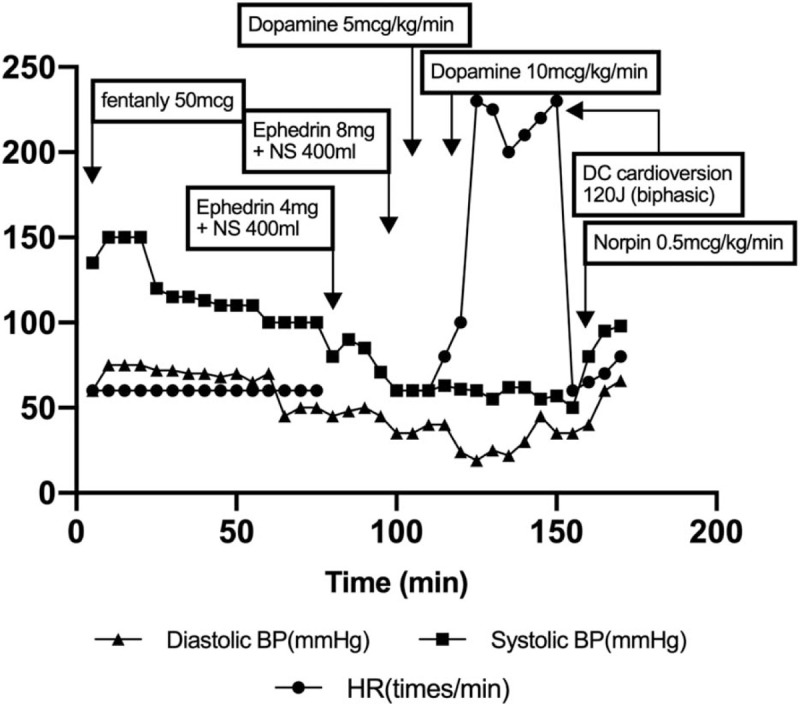
Vital signs at the PACU.

At the time of transfer from the operating room to PACU, the patient had a noninvasively measured blood pressure (NIBP) of 138/60 mmHg, HR of 60 beats/min, and temperature of 35.5°C. Immediately after entering the PACU, the patient complained of sharp throbbing pain at the surgical site (abdomen) with an numeral rating scale score (NRS) of 8 points and received 50 mcg of fentanyl. Subsequently blood pressure gradually decreased. Due to the delay in transfer to the general ward, the patient stayed in the PACU for a long time. Ninety minutes after PACU admission, NIBP dropped to 80/45 mmHg. To increase the blood pressure, 400 mL of fluid and 4 mg of ephedrine were administered, but the blood pressure did not increase, and the patient was sweating; however, he was mentally alert. The NRS score in the surgical area dropped from 8 to 3 points. Furthermore, 400 mL of fluid and 8 mg of ephedrine were administered again, but blood pressure remained low. After 20 minutes, NIBP decreased to 60/40 mmHg. There were no skin rashes or symptoms of allergy (Fig. 2).

Continuous dopamine infusion was started at a rate of 5 mcg/kg/min with fluid therapy; however, after 10 minutes, blood pressure reduced to 60/35 mmHg, and dopamine infusion rate was increased to 10 mcg/kg/min. Ten minutes after infusion, NIBP was still at 61/19 mmHg, and HR suddenly increased to 200 to 230 beats/min. EKG was performed, and monomorphic VT was observed.

**Figure 3:**
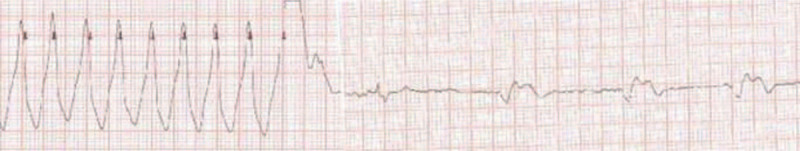
DC cardioversion (120J biphasic); monomorphic VT converse to sinus rhythm after DC cardioversion on Defibrillator EKG. HR decreased from 200 beats/min to 60 beats/min. After DC cardioversion, patient's self heart rhythm ceased and pacemaker pace at 60 beasts/min.

The patient was alert but sweating, and his femoral pulse was palpable. Monomorphic VT with pulse persisted and the patient was in sinus rhythm for 10 second intermittently. However, within a few seconds, HR again increased to 200 beats/min, and monomorphic VT with pulse continued. Arterial cannulation was performed. After administration of 2 mg of midazolam, DC cardioversion 120 J (biphasic) was performed. HR was maintained at 60 beats/min, arterial blood pressure (ABP) was 50/41 mmHg after DC cardioversion, and continuous infusion of norepinephrine was started at a rate of 0. 5 mcg/kg/min. (Fig. 3) in approximately 7 minutes, the patient recovered with an ABP of 95/60 mmHg and HR of 80 to 90 beats/min; the patient showed improved symptoms, was nearly mentally alert, and was sweating less.

**Figure 4:**
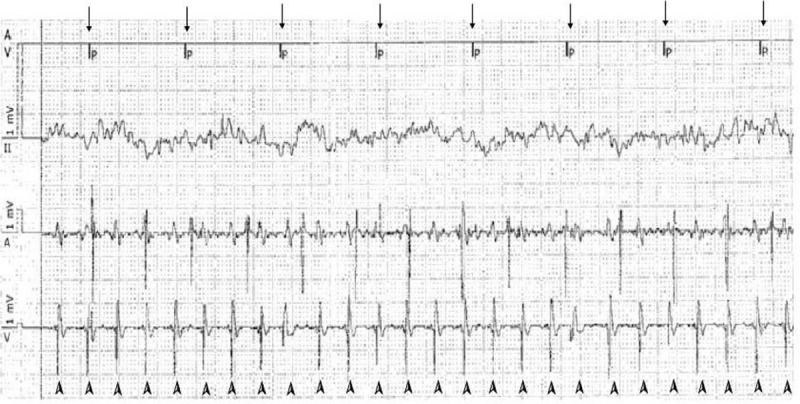
Immediate PM interrogation result with monomorphic ventricular tachycardia (DOO mode). PM pacing the ventricle with a rate of 60 beats/min, which we could exclude pacemaker-mediated-tachycardia, and malfunction of PM. Arrow marks show a pacemaker pacing. Also we can see the VT on pacemaker interrogation with HR of 200 beats/min. Arrow head marks show ventricle contraction at a rate of 200 beats/min. (monomorphic VT).

**Figure 5:**
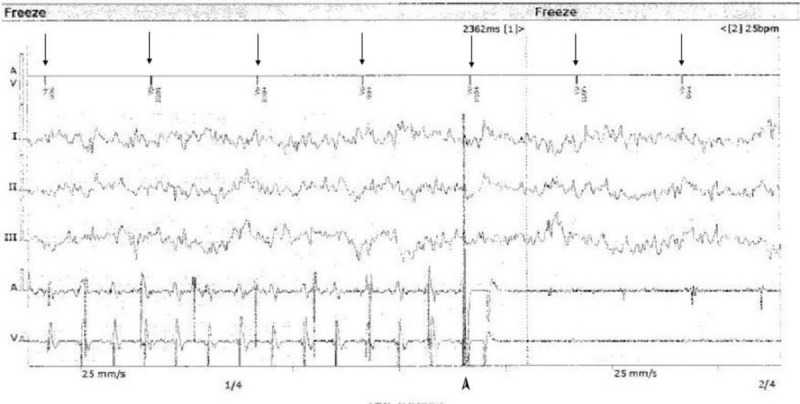
Pacemaker interrogation result at the time of DC cardioversion. Ventricular pacing with HR at 60 beats/min is maintained, and monomorphic VT ceased with DC cardioversion. Arrow marks show PM pacing at a rate of 60 beats/min. DC cardioversion (biphasic 120 J) was executed and arrhythmia ceased. Arrow head mark shows at the time of DC cardioversion.

Immediate pacemaker interrogation was performed, but no abnormalities in the pacemaker were observed. The pacemaker had a pacing rate of 60 beats/min, which eliminated pacemaker-mediated tachycardia (Figs. 4 and 5). The pacemaker mode was changed from DOO (asynchronous mode) to DDDR, and the patient was transferred to the intensive care unit (ICU) for observation. The total fluid administered at the PACU was 1000 mL and ABGA showed pH of 7. 31, PCO2 of 42. 0 mmHg, PO2 of 97. 6 mmHg, sodium level of 143 mEq/L, potassium level of 4. 0 mEq/L, calcium level of 7. 2 mg/dL and an Hgb level of 10. 4 g/dL. The total urine output at the PACU was 100 mL.

A 12-lead EKG which was recorded in the ICU showed no PVCs, and portable transthoracic echocardiography revealed no specific findings.

One month later, the patient visited the emergency room (ER) with symptoms of dizziness, low blood pressure (systolic blood pressure of 80–90 mmHg), and presyncope. EKG at the ER showed AV dual-paced rhythm and frequent PVC with tall R waves in leads II, III, and aVF, which was the same as frequent PVC derived from the RVOT observed previously. QTc was 495 ms. The patient recovered after supportive care, including fluid loading, and was discharged.

### 2.1 Ethics statement

This case report was approved and registered by the Institutional Review Board of Inha university hospital (IRB No. 2020–01–005). There was no potential conflict of interest relevant to this article. The patient has provided informed consent for publication of the case.

## 3 Discussion

Newly developed postoperative arrhythmias are reported in 10% to 40% of patients undergoing cardiothoracic surgery and 4% to 20% of those undergoing non-cardiac surgery.^[3]^ Most cases of arrhythmia during or after non-cardiac surgery occur in patients who undergo major abdominal or vascular surgery. Nonvascular abdominal surgery itself is an independent risk factor for arrhythmia. Most arrhythmias are supraventricular and include atrial fibrillation (4. 41%), atrial flutter (0. 94%), paroxysmal atrial tachycardia (0.3%), multifocal atrial tachycardia (0.4%), and paroxysmal supraventricular tachycardia (3%), and Ventricular arrhythmias are rare. ^[3,4]^ The etiology of these arrhythmias comprises stress responses due to surgery and anesthetic procedures, which increase sympathetic and hormonal activity and enhance systemic inflammatory pathways. The prognosis for newly developed postoperative arrhythmias is favorable, as most of the cases are self-limiting and return to the original heart rhythm occurs in approximately 80% of cases.^[5]^

Idiopathic VT, which occurs in individuals without structural abnormalities in the heart, accounts for approximately 10% of all cases of VT, and the most common site of origin of VT is the RVOT.^[6]^ Approximately 70% of idiopathic VTs originate from the RVOT.^[6]^ This area is not only the site of origin of VT but also of PVCs. It has unique morphological characteristics in the EKG, consisting of LBBB with inferior axis, right axis deviation on standard limb leads, and early precordial transition, which can be recognized in the 12-lead EKG. (Fig. 1) The RVOT has a complex structure and it is structurally adjacent to the aortic valve, mitral valve, and left ventricular outflow tract. As a developmental feature, the distribution of connexin is low, resulting in weak intercellular connections, slow conduction rate, and no contractile force.^[7]^ These developmental characteristics affect the conduction velocity and refractory period in myocardial cells, triggering delayed depolarization that leads to sustained Ca^2+^ outflow from the sarcoplasmic reticulum at phase 4 of repolarization in myocardial cells, and activation of the 3Na^+^/Ca^2+^ exchanger. These actions eventually increase intracellular Ca^2+^ concentration. In this process, “cAMP-mediated triggered activity” by adenylyl cyclase (AC) is important for the initiation and termination of tachycardia.^[8]^

Acceleration of HR can be developed by programmed ventricular stimulation, burst pacing, infusion of a catecholamine or exercise. This is why termination is possible by the administration of adenosine, a beta-blocker, a calcium channel blocker and by vagal maneuvers.^[9,10]^ (Table 1).

**Table 1:** Ventricular tachycardia triggering factors and its treatments.

VT triggering factor	Treatment
Isoproterenol	Adenosine
Exercise	Verapamil
Rapid burst pacing, atropine	Vagal maneuvers (acetylcholine)
Aminophylline	Beta adrenergic receptor blockade

The patient also had a typical EKG pattern of PVC from the RVOT. Patient's vital sign and hemodynamic status was stable during surgery. However patient's blood pressure gradually decreased after pain control and EKG monitoring is not routinely performed at PACU. Although it was before EKG monitoring, it is highly likely that patient had low blood pressure, which was most likely due to decreased preload and PVCs. Patient's history of repeat visit to ER also strongly support this basis. Ephedrine, dopamine which had led on β_1_ adrenergic receptors agonist effects and increased activity of AC resulting in “cAMP-mediated triggered activity,” which could culminate in VT.^[8,9,11]^ Unfortunately, in this case, since the patient initially had stable hemodynamic indices, only NIBP and pulse oximetry monitoring were performed, and EKG monitoring was performed after the vital signs became unstable at the PACU.

Considering the relatively prolonged QTc (499 ms) in the patient's initial EKG, postoperative PVCs might have occurred during the vulnerable period of the central 1/3 of the T-wave on EKG leading polymorphic VT rather than monomorphic VT.^[12]^ Moreover, in this patient, pacemaker interrogation reveals that there was no triggering activity by DOO mode, but if the DDD mode of the dual-chamber pacemaker with ventriculoatrial (retrograde) conduction exists, PVCs could induce retrograde conduction that leads to “endless loop tachycardia,” resulting in “pacemaker-mediated tachycardia.” In such a scenario, monomorphic VT is possible.^[13]^ These 2 scenarios that could cause VT did not occurred and could be ruled out.

Idiopathic ventricular outflow tachycardia has 3 clinical forms: paroxysmal sustained monomorphic VT, repetitive non-sustained VT, or PVCs. Its symptoms are chest pain (13%), palpitations (39.8%), dizziness (3.7%), dyspnea (3.1%) and syncope (2.5%), and sometimes, it is asymptomatic (37.9%).^[14,15]^ Even in this patient, there is a possibility of arrhythmia may cause Postoperation hypotension at the PACU. Another possibility is that the blood loss (400 mL) and the urine output (630 mL), together with the modest total volume of fluid replacement of 2100 mL, resulted in a relative hypovolemic state. Additionally, the patient repeatedly visited the ER with presyncope symptoms of dizziness and low blood pressure, and EKG at the ER also revealed frequent PVCs from the RVOT. This likely reflects that decrease in intravascular volume may led to frequent PVCs originating from RVOT and led to hypotension with patient. Interrogation with patient's pacemaker also showed that 7% of all heartbeats were PVCs which strongly support this idea.

In conclusion, we report a case of frequent PVCs from the RVOT after colon cancer surgery in a SSS patient with a permanent pacemaker, which led to VT by triggering activity. In this situation, patient's blood pressure was decreased first. At this time, not only we empirically administrate fluids but also need immediate EKG monitoring. In order to increase blood pressure, fluid resuscitation and direct acting vasoconstrictors like norepinephrine might be a better drug of choice. It is recommended to avoid ephedrine or dopamine, which may cause ventricular tachycardia due to “cAMP-mediated triggering activity” with patient who has frequent PVCs from RVOT. Furthermore, monomorphic VT is possible in patients with frequent PVC originating from the RVOT, and active pain control and fluid resuscitation should be considered with simple hemodynamic monitoring like EKG, PVI, and Pi during the perioperative period and at the PACU. Moreover, in a case of drug-resistant VT and recurrent VT, active treatment such as radiofrequency ablation could be an option.

## Author contributions

**Conceptualization:** Na Eun Kim, Yong Soo Baek, Helen Ki Shinn.

**Data curation:** Yong Soo Baek.

**Formal analysis:** Hyun Kyoung Lim.

**Investigation:** Na Eun Kim.

**Resources:** Yong Soo Baek, Helen Ki Shinn.

**Supervision:** Hyun Kyoung Lim.

**Writing – original draft:** Kihyun Park, Na Eun Kim, Yong Soo Baek.

**Writing – review & editing:** Kihyun Park, Hyun Kyoung Lim, Helen Ki Shinn.
